# Letter to the editor: social influences on the relationship between dissociation and psychotic-like experience

**DOI:** 10.1017/S0033291724002277

**Published:** 2024-11

**Authors:** Hisao Toyoshima, Tomohide Akase

**Affiliations:** 1 Pharmacy Management Institute, Graduate School of Business, Japan University of Economics, Shibuya-ku, Tokyo, Japan; 2 Graduate School of Business, Japan University of Economics, Shibuya-ku, Tokyo, Japan

We read with great interest the paper by Charles Heriot-Maitland, Til Wykes, and Emmanuelle Peters titled ‘Social influences on the relationship between dissociation and psychotic-like experiences’ (*Psychological Medicine*, 1–9, 2024). In this study, social factors were extracted from experiences such as feeling humiliated by others and feelings of safety and connection with others. However, we believe there are several limitations and issues that warrant attention. Here are some examples.

1. The conclusion that ‘two factors (shame and psychological safety) may be involved in psychotic-like experiences’ may be overly definitive. Reports indicate that the onset of psychotic-like experiences (PLE) involves factors beyond social influences such as shame and low psychological safety environments. These include a history of psychotic disorders (including medication use [Linscott & van Os, 2013]), sleep (Freeman et al., 2017), genetic factors (Legge et al., 2019), unemployment, leave of absence (Stilo et al., 2013), economic difficulties, and cultural factors (Zandi et al., 2010). Without conducting a survey that considers these factors, it is difficult to conclusively determine the involvement of only shame and psychological safety. Therefore, further examination is necessary, including the possibility that these factors may be confounding variables.
2. In the examination of models A, B, and C, the temporal order of shame's influence on dissociation is unclear. The study design does not allow for distinguishing whether shame preceded and influenced dissociation, or if shame resulted from dissociation. A more detailed explanation of this point is required.
3. The measurement periods for each score are not uniform, potentially affecting the quality and reliability of the data. PLE were measured over 1 week, the period for the Dissociative Experiences Scale (DES) is unclear, the Other as Shamer scale (OAS) over 2 weeks, and the Social Safeness and Pleasure Scale (SSPS) over 1 week. This variability in measurement periods may increase respondent bias, necessitating caution in generalizing results to all subjects (Stull, Leidy, Parasuraman, & Chassany, 2009).
4. While the study uses a 6-month follow-up for PLE in the longitudinal study, reviews suggest that at least 12 months of follow-up is necessary for proper evaluation (Alvarez-Jimenez et al., 2012). Therefore, medium- and long-term examinations are needed.
5. While sampling from the general population is useful, attention needs to be paid to cultural sensitivity, cross-country comparisons, consideration of cultural contexts, and the use of mixed research methods (Linscott & van Os, 2013).

Considering these points, we suggest that the authors make the following additions to points 1–5:

For point 1, provide detailed explanations of the subjects’ history of psychotic disorders (including medication use), sleep status, genetic factors, employment status, economic situation, and cultural background. If these factors were excluded, present the results of their impact assessment.

For point 2, provide a detailed explanation of the temporal relationship between dissociation and shame in the model.

For point 3, acknowledge the implications of different time scales for each measurement scale and specify precautions for accurately assessing emotional states and their duration. Consider using scales that evaluate a 1-week period, such as the Experience of Shame Scale (ESS).

For point 4, extend the follow period to 12–24 months or longer and increase measurement points to more appropriately assess the long-term course of PLE and their association with social factors. Modify the conclusion to state, ‘The 6-month observation suggested a possible association between social factors and PLE reporting, but further comprehensive research is needed to elucidate long-term stability and causal relationships’, to appropriately convey the study's limitations and significance of findings. For point 5, consider using data from both general population and clinical samples, as it has been suggested that this approach allows for a more comprehensive understanding of the process of psychosis onset, thereby deepening the significance of the findings.

We believe that these responses improved the quality and validity of this study. Thank you for considering our manuscript.
